# Association between a family history of diabetes and carotid artery atherosclerosis in Korean adults

**DOI:** 10.4178/epih.e2021049

**Published:** 2021-08-03

**Authors:** Sun Young Shim, Ga Bin Lee, Jee-Seon Shim, Sun Jae Jung, Hyeon Chang Kim

**Affiliations:** 1 Department of Public Health, Graduate School, Yonsei University, Seoul, Korea; 2 Cardiovascular and Metabolic Diseases Etiology Research Center, Yonsei University College of Medicine, Seoul, Korea; 3 Department of Preventive Medicine, Yonsei University College of Medicine, Seoul, Korea

**Keywords:** Diabetes mellitus, Atherosclerosis, Carotid intima-media thickness, Medical history taking

## Abstract

**OBJECTIVES:**

Diabetes is a well-known risk factor for atherosclerosis, but the association between a family history of diabetes and atherosclerosis remains unknown. In this study, we assessed the association between a family history of diabetes and increased carotid intima-media thickness (IMT), a marker of subclinical atherosclerosis, in a middle-aged Korean population.

**METHODS:**

This cross-sectional study included 3,974 community-dwelling adults (1,404 male and 2,570 female) aged 30-64 years from the Cardiovascular and Metabolic Diseases Etiology Research Center cohort. The presence of a family history of diabetes was assessed through face-to-face interviews using a standardized questionnaire. Carotid IMT was assessed using B-mode ultrasonography, and increased IMT was defined as a value in the top quartile of the IMT values of all participants. Multivariate logistic regression was used to evaluate independent associations between a family history of diabetes and increased IMT.

**RESULTS:**

A family history of diabetes was significantly associated with increased carotid IMT (odds ratio, 1.23; 95% confidence interval, 1.03 to 1.48) after adjusting for sex; age; body mass index; systolic blood pressure; total cholesterol, triglyceride, and hemoglobin A1c levels; smoking; alcohol consumption; exercise; use of antidiabetic, antihypertensive, and antilipidemic drugs; and a family history of hypertension. The positive association remained significant after excluding participants with diabetes (odds ratio, 1.21; 95% confidence interval, 1.00 to 1.47).

**CONCLUSIONS:**

A family history of diabetes was positively associated with increased carotid IMT, even in participants without diabetes. Therefore, information on a family history of diabetes may help identify individuals at high risk of atherosclerotic cardiovascular disease.

## INTRODUCTION

Approximately 415 million people worldwide have diabetes mellitus, and an estimated 193 million people have undiagnosed diabetes [1]. Diabetes is a primary risk factor for atherosclerosis [2,3], stroke [4], coronary heart disease [5], and dyslipidemia [6]. People with diabetes have a higher prevalence and/or greater severity of atherosclerosis than those without diabetes [7]. This predisposition to severe atherosclerosis considerably contributes to increased morbidity and mortality from coronary artery disease in patients with diabetes [7]. The link between diabetes and the risk of atherosclerotic cardiovascular disease has been widely reported in the literature [2,7-10]. However, only a few studies have assessed the association between a family history of diabetes and atherosclerosis.

Individuals with a family history of diabetes have a higher risk of diabetes than persons without a family history of diabetes [11]. A family history of diabetes has also been associated with cardiometabolic disorders that can trigger and modify the atherosclerotic process [12]. However, inconsistent results have been reported regarding whether a family history of diabetes is related to atherosclerosis independent of other risk factors [13-15]. Some studies have shown that a family history of type 2 diabetes mellitus is significantly associated with the risk of atherosclerosis among individuals without diabetes [15,16], whereas other works have not identified such a link [13,17].

Clarifying these inconclusive results is important because information on a family history of diabetes might be a useful tool to identify individuals at risk of atherosclerotic cardiovascular disease [18,19]. Therefore, this study aimed to assess the association between a family history of diabetes and increased intima-media thickness (IMT) of the carotid arteries, a marker of general subclinical atherosclerosis, in a middle-aged Korean population. We hypothesized that participants with a family history of diabetes would be more likely to have increased carotid IMT than those without a family history of diabetes.

## MATERIALS AND METHODS

### Study population

This cross-sectional study analyzed baseline data from a research clinic at the Cardiovascular and Metabolic Diseases Etiology Research Center (CMERC). We excluded 36 people who answered that they were uncertain whether either of their parents had diabetes, and 50 participants without IMT measurements or covariates. In total, 3,974 community-dwelling adults (1,404 male and 2,570 female) aged 30-64 years, without myocardial infarction, stroke, or heart failure, completed a questionnaire survey and underwent regular health examinations [20].

### Measurements

A family history of diabetes was assessed through face-to-face interviews using standardized questionnaires following the pre-established CMERC protocol [21]. Participants had to report whether a family member (father, mother, or sibling) had been diagnosed with or died from a specific disease [21]. The primary analysis was conducted only on the participants who knew whether their parents had diabetes. A family history of diabetes was defined as having a father or mother who had been diagnosed with or died from diabetes. We additionally included data from participants who were unsure whether they had a family history of diabetes in a sensitivity analysis. In this analysis, parents who were uncertain of whether they had diabetes were considered not to have diabetes. Participants with diabetes were defined as individuals with a previous diagnosis of diabetes by a medical doctor, a fasting plasma glucose level ≥ 126 mg/dL, or hemoglobin A1c (HbA1c) ≥ 6.5%. Impaired fasting glucose was defined as a fasting plasma glucose level of 100-125 mg/dL.

Carotid arteries were examined using B-mode ultrasonography (Accuvix XG; Samsung Medison, Seoul, Korea), which was performed by trained operators according to a predefined study protocol [21]. Carotid IMT was measured at the 1-cm segment of the common carotid arteries proximal to the carotid bulb region, at the time of the R-wave on the electrocardiogram, and was performed bilaterally using dedicated software [21]. The interrater reliability of IMT measurements was high [22]. Increased IMT was defined as a mean carotid IMT on the left or right sides greater than or equal to the cut-off value (≥ 0.717 mm). The cut-off value was set to the larger of each of the highest quartile values of the mean carotid IMT measured on both sides. Sex-specific cut-off levels were used for a sex-stratified analysis (male: ≥ 0.726 mm, female: ≥ 0.710 mm).

As for the covariates, physical activity was assessed using the International Physical Activity Questionnaire-Short Form [23,24], and regular exercise was defined as moderate or high-intensity physical activity more than twice a week [25]. Participants were classified by smoking and alcohol intake status as current smokers and former/never smokers, and current drinkers and former/never drinkers, respectively. Medication status was assessed using a questionnaire and divided into currently taking drugs and not taking drugs. A family history of hypertension was defined as having a parent with a hypertension diagnosis or who died from hypertension-related causes.

Body weight was measured to the nearest 0.1 kg using a digital scale (DB-150; CAS, Seoul, Korea), and standing height was measured to the nearest 0.1 cm using a stadiometer (DS-102; JENIX, Seoul, Korea). Body mass index (BMI) was calculated as the body weight divided by height squared (kg/m^2^) and classified into the following groups: < 23 kg/m^2^, 23 kg/m^2^ to < 25 kg/m^2^, 25 kg/m^2^ to < 30 kg/m^2^, and ≥ 30 kg/m^2^ [26]. Blood pressure was measured with the participants in a sitting position and after they had rested for at least 5 minutes using an automated oscillometric device (HEM-7080; Omron Healthcare, Kyoto, Japan). Systolic and diastolic blood pressure measurements were made 3 times each at 2-minute intervals in the right arm [21], and the mean of the last 2 measurements was used in the analysis. Overnight fasting blood samples were collected from all participants by a trained researcher from the antecubital vein. Detailed information about the measurements in the CMERC study has been published elsewhere [21].

### Statistical analysis

We evaluated the differences in variables according to a family history of diabetes using the 2-sample t-test or Mann-Whitney U test for continuous variables and the chi-square test for categorical variables. The association between a family history of diabetes and increased carotid IMT was analyzed using multivariate logistic regression models adjusted for sex; age; BMI; systolic blood pressure; total cholesterol, triglyceride, and HbA1c levels; smoking and drinking status; regular exercise; use of antihypertensive, lipidlowering, and antidiabetic drugs; and a family history of hypertension. These analyses were performed on 3,974 participants and repeated on 3,691 participants after excluding 283 people with diabetes, and on 3,244 participants after additionally excluding 447 people with impaired fasting glucose. The same multivariate logistic regression model was used to examine the association between a family history of diabetes, including those with an unknown family history, and increased carotid IMT. Lastly, the associations in subgroups defined according to sex, age, BMI, smoking status, hypertension, or dyslipidemia were investigated. The putative interactions within each subgroup were included in the model and tested. Effect sizes were reported using odds ratios (ORs) with 95% confidence intervals (CIs) and p-values. All statistical tests were performed using SAS version 9.4 (SAS Institute Inc., Cary, NC, USA).

### Ethics statement

The study protocol was approved by the Institutional Review Board (IRB) of Severance Hospital at Yonsei University College of Medicine (IRB No. 4-2013-0661). Informed consent was confirmed by the IRB. All procedures in this work complied with the ethical standards of the relevant national and institutional committees on human experimentation and the Helsinki Declaration of 1975, as revised in 2008.

## RESULTS

### General characteristics

Table 1 summarizes the characteristics of the study participants based on the presence of a family history of diabetes. The sex-stratified data are provided in Supplementary Material 1. Of the 3,974 individuals, 903 (22.7%) had a family history of diabetes. These participants had higher BMI, fasting glucose, and HbA1c levels, but lower high-density lipoprotein cholesterol levels, than participants without a family history of diabetes. There was no difference in the mean carotid IMT between people with a family history of diabetes and those without (0.679 mm and 0.675 mm, respectively; p=0.378) because a family history of diabetes was more frequently reported among younger participants. The ageadjusted carotid IMT was significantly higher in participants with a family history of diabetes than those without (0.685 mm and 0.676 mm, respectively; p=0.021). A family history of hypertension and myocardial infarction, antidiabetic medication, and diagnosis of diabetes were more frequent among people with a family history of diabetes. This result was also founded in the sex-stratified analysis (Supplementary Material 1).

### Association between a family history of diabetes and carotid atherosclerosis

In all participants, a family history of diabetes was positively and independently associated with increased IMT (OR, 1.23; 95% CI, 1.03 to 1.48; p=0.023), after adjusting for sex; age; BMI; systolic blood pressure; total cholesterol, triglyceride, and HbA1c levels; smoking and drinking status; regular exercise; use of antihypertensive, lipid-lowering, and antidiabetic drugs; and a family history of hypertension (Table 2). The association remained significant even after excluding individuals with diabetes (OR, 1.21; 95% CI, 1.00 to 1.47; p=0.048). A similar association was also observed after additionally excluding people with impaired fasting glucose (OR, 1.21; 95% CI, 0.98 to .48).

**Figure 1.**
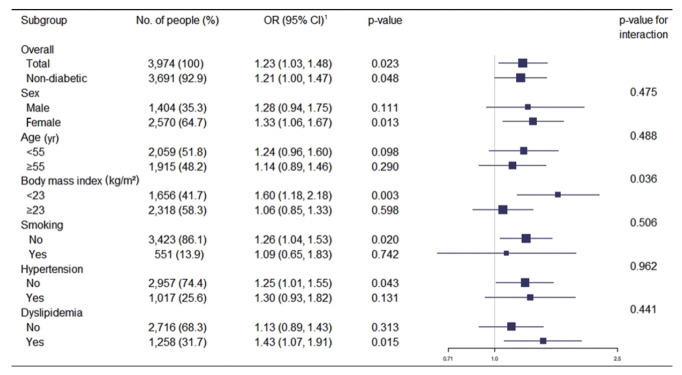
Associations between a family history of diabetes and increased carotid intima-media thickness in different subgroups. OR, odds ratio; CI, confidence interval. ^1^Adjusted for all other factors not involving the subgroup, including sex, age, body mass index, systolic blood pressure, total cholesterol, triglyceride, hemoglobin A1c, smoking status, drinking status, regular exercise, antihypertensives, lipid-lowering drug, antidiabetics and family history of hypertension.

Figure 1 shows the association between a family history of diabetes and increased IMT in various subgroups. After adjusting for covariates, the OR for the multivariate logistic regression analysis was 1.28 (p=0.111) in males and 1.33 (p=0.013) in females. A family history of diabetes was positively associated with increased IMT in most subgroups, although the strength of the association varied. The ORs for increased carotid IMT were particularly high for participants with a BMI < 23 kg/m^2^ (OR, 1.60; 95% CI, 1.18 to 2.18), non-smokers (OR, 1.26; 95% CI, 1.04 to 1.53), those without hypertension (OR, 1.25; 95% CI, 1.01 to 1.55), and those with dyslipidemia (OR, 1.43; 95% CI, 1.07 to 1.91). The strength of the association was significantly different between participants with a BMI ≥ 23 kg/m^2^ and those with a BMI < 23 kg/m^2^ (p for interaction= 0.036). No significant differences were observed between the other subgroups.

In a sensitivity analysis that included participants who did not know whether either of their parents had diabetes, the association between a family history of diabetes and increased IMT persisted for all participants (OR, 1.25; 95% CI, 1.05 to 1.50) and for the participants without diabetes (OR 1.24; 95% CI 1.03 to 1.50) (Table 3).

## DISCUSSION

We found a significant positive association between a family history of diabetes and increased carotid IMT in participants with and without diabetes while controlling for potential confounders.

Only a few studies have demonstrated this association. An analysis of 620 individuals without diabetes from 24 Mexican-American families reported a significant association between a family history of type 2 diabetes and increased common carotid artery IMT [14]. A cross-sectional study of 401 people with normal glucose tolerance reported a significant association between a family history of type 2 diabetes and increased IMT [15]. In a case-control study, the first-degree offspring of patients with type 2 diabetes had reduced total insulin sensitivity and impaired beta-cell function, both of which were related to increased internal carotid artery IMT, compared to individuals without a family history of diabetes [16]. However, conflicting results have also been reported in the literature. A cross-sectional study showed that carotid IMT was higher in a subgroup of participants with high low-density lipoprotein cholesterol (LDL-C) and a family history of cardiovascular disease than in individuals without diabetes who had a family history of diabetes [13]. Another study involving 6,434 participants who had undergone health examinations showed that a family history of diabetes was not significantly associated with the development of subclinical atherosclerosis in participants without diabetes after adjusting for clinical variables. However, the association was significant in individuals with diabetes [17]. This study suggests that the combination of chronic hyperglycemia and a family history of diabetes might be critical in the development of atherosclerosis.

Given that people with a family history of diabetes often do not receive screening for diabetes or risk factors, it is important to understand how a family history of diabetes contributes to the development of cardiovascular disease [27]. A previous report showed that participants without diabetes, but with a family history of diabetes, had a higher risk of glucose intolerance [28] and lower insulin sensitivity [13]. Hyperglycemia causes non-enzymatic glycosylation of proteins, leading to the production of advanced glycation products, which can damage the arterial walls and possibly contribute to atherosclerosis [29]. In addition, a family history of diabetes is related to abnormal blood lipid levels [13] and the accumulation of LDL-C in the subendothelial matrix; this pattern may be linked to atherosclerosis, since elevated LDL-C levels are characteristic of early atherosclerosis [29]. Thus, impaired glucose and lipid metabolism may lead to atherosclerosis in individuals with a family history of diabetes. However, alternative explanations are indispensable because the independent association between a family history of diabetes and increased IMT persisted after adjusting for HbA1c, total cholesterol, and triglyceride levels. This independent association could be related to endothelial dysfunction [30], impaired fibrinolysis [31], as yet unidentified genetic factors, inflammatory processes [14], or interactions or mediating effects not considered in our analysis. A previous study suggested that elevated fasting and postprandial glucose concentrations in the offspring of patients with type 2 diabetes could lead to the loss of endothelial function, resulting in increased IMT [15]. Another explanation is that pre-existing inflammation before impaired glucose metabolism triggers arteriosclerosis [14].

This study has some limitations. First, it may have been affected by recall bias, as participants self-reported their family history of diabetes. However, in a validation study comparing parental history reported by the offspring with parents’ confirmed medical history, positive and negative reports of a parental history of diabetes were found to be reliable [32]. Nevertheless, misclassification cannot be ruled out because there are no available data on the validity of the family history questionnaire in our cohort. The second limitation concerns operator dependency, which is a well-known problem in ultrasonographic measurements. Since carotid IMT was calculated by assessing the carotid arteries using B-mode ultrasonography, there may have been inconsistencies between operators and devices [33]. Although we followed a consistent protocol for our measurements and used an identical instrument at a single health center, variance in measurements between technicians could not be completely excluded. Third, our analysis did not include data from oral glucose tolerance tests to define impaired glucose tolerance. Impaired glucose tolerance and impaired fasting glucose differ in terms of the insulin resistance site [34]. However, both pre-diabetic conditions reflect intermediate states of abnormal glucose control that exist between diabetes and normal glucose homeostasis [34]. Fourth, although we included well-known risk factors for atherosclerosis in the adjusted models, there may have been residual confounding that we did not control for. Lastly, the individuals in this cohort were not randomly selected and they did not represent the entire Korean population. Thus, the findings of our study may not be generalizable to other populations.

In conclusion, there was a positive association between a family history of diabetes and increased carotid IMT. This association was significant even in individuals without diabetes. Our findings suggest that checking for a family history of diabetes may help identify individuals at high risk of atherosclerotic cardiovascular disease. To deepen our understanding of these results, further studies involving a large number of participants, hereditary research, prospective cohorts, and meta-analyses are needed.

## Figures and Tables

**Table 1.** General characteristics of the study participants according to the presence of a family history of diabetes

Characteristics	Family history of diabetes		p-value
	No (n=3,071)	Yes (n=903)	
Age (yr)	51.8±9.3	49.6±9.2	<0.001
Female	1,997 (65.0)	573 (63.5)	0.407
Body mass index (kg/m^2^)	23.8±3.0	24.2±3.1	0.002
Systolic blood pressure (mmHg)	118.6±15.1	118.3±14.2	0.570
Diastolic blood pressure (mmHg)	76.1±10.0	76.3±9.6	0.592
Total cholesterol (mg/dL)	198.6±34.8	198.6±37.5	0.957
HDL cholesterol (mg/dL)	57.9±14.7	56.6±14.8	0.018
LDL cholesterol (mg/dL)	118.7±31.8	119.1±33.7	0.759
Triglyceride (mg/dL)	106 [76-150]	109 [79-160]	0.049
Fasting glucose (mg/dL)	88 [82-95]	90 [83-98]	<0.001
Hemoglobin A1c (%)	5.64±0.65	5.78±0.82	<0.001
Hypertension	806 (26.3)	211 (23.4)	0.089
Dyslipidemia	942 (30.7)	316 (35.0)	0.016
Diabetes	184 (6.0)	99 (11.0)	<0.001
Family history of hypertension	984 (32.0)	501 (55.5)	<0.001
Family history of cardio-cerebrovascular disease			
Stroke	456 (14.9)	133 (14.7)	0.969
Myocardial infarction	174 (5.7)	93 (10.3)	<0.001
Medication uses			
Antihypertensives	485 (15.8)	120 (13.3)	0.074
Lipid-lowering drugs	349 (11.4)	108 (12.0)	0.664
Antidiabetics	110 (3.6)	74 (8.2)	<0.001
Health behaviors			
Current smoker	420 (13.7)	131 (14.5)	0.562
Current drinker	2,233 (72.7)	686 (76.0)	0.057
Regular exercise	1,088 (35.4)	334 (37.0)	0.412
Carotid IMT (mm)^1^	0.679±0.123	0.675±0.120	0.378
Age-adjusted carotid IMT (mm)	0.676 ±0.002	0.685±0.004	0.021

Values are presented as mean ± standard deviation, median [interquartile range], or number (%). HDL, high-density lipoprotein; LDL, low-density lipoprotein; IMT, intima-media thickness.

1 The larger of the right mean carotid IMT and left mean carotid IMT was determined as the carotid IMT.

**Table 2.** Factors associated with increased carotid IMT

Variables		All participants (n=3,974)			Participants without diabetes (n=3,691)			Participants without diabetes or impaired fasting glucose (n=3,244)		
		No. of people	People with an increased IMT, n (%)	OR (95% CI)	No. of people	People with an increased IMT, n (%)	OR (95% CI)	No. of people	People with an increased IMT, n (%)	OR (95% CI)
FH of diabetes										
	No	3,071	1,070 (34.8)	1.00 (reference)	2,887	974 (33.7)	1.00 (reference)	2,553	817 (32.0)	1.00 (reference)
	Yes	903	315 (34.9)	1.23 (1.03, 1.48)	804	264 (32.8)	1.21 (1.00, 1.47)	691	210 (30.4)	1.21 (0.98, 1.48)
Sex										
	Female	2,570	857 (33.4)	1.00 (reference)	2,434	792 (32.5)	1.00 (reference)	2,224	691 (31.1)	1.00 (reference)
	Male	1,404	528 (37.6)	1.08 (0.90, 1.30)	1,257	446 (35.5)	1.03 (0.85, 1.25)	1,020	336 (32.9)	0.97 (0.78, 1.20)
Age (per 10 yr)		-	-	2.81 (2.52, 3.13)	-	-	2.81 (2.51, 3.15)	-	-	2.96 (2.61, 3.35)
BMI (kg/m^2^)										
	<23	1,656	433 (26.2)	1.00 (reference)	1,604	406 (25.3)	1.00 (reference)	1,490	362 (24.3)	1.00 (reference)
	23-<25	1,007	364 (36.2)	1.26 (1.05, 1.52)	938	330 (35.2)	1.27 (1.05, 1.54)	823	286 (34.8)	1.35 (1.10, 1.66)
	25-<30	1,172	524 (44.7)	1.79 (1.49, 2.16)	1,038	452 (43.6)	1.82 (1.49, 2.21)	846	343 (40.5)	1.75 (1.42, 2.17)
	≥30	139	64 (46.0)	2.12 (1.41, 3.19)	111	50 (45.1)	2.28 (1.44, 3.59)	85	36 (42.4)	2.06 (1.23, 3.46)
SBP (per 10 mmHg)		-	-	1.19 (1.13, 1.25)	-	-	1.18 (1.12, 1.25)	-	-	1.19 (1.12, 1.26)
Total cholesterol (per 10 mg/dL)		-	-	1.02 (1.00, 1.04)	-	-	1.02 (1.00, 1.04)	-	-	1.02 (0.99, 1.04)
Triglyceride (per 10 mg/dL)		-	-	1.00 (0.99, 1.01)	-	-	1.00 (0.99, 1.01)	-	-	1.01 (0.99, 1.02)
Hemoglobin A1c (per 1.0%)		-	-	1.23 (1.09, 1.39)	-	-	1.42 (1.14, 1.76)	-	-	1.17 (0.88, 1.57)
Smoking status										
	Non/former	3,423	1,213 (35.4)	1.00 (reference)	3,195	1,093 (34.2)	1.00 (reference)	2,825	918 (32.5)	1.00 (reference)
	Current	551	172 (31.2)	1.22 (0.95, 1.56)	496	145 (29.2)	1.26 (0.97, 1.64)	419	109 (26.0)	1.22 (0.91, 1.63)
Drinking status										
	Non/former	1,055	412 (39.1)	1.00 (reference)	976	374 (38.3)	1.00 (reference)	880	331 (37.6)	1.00 (reference)
	Current	2,919	973 (33.3)	0.84 (0.71, 0.99)	2,715	864 (31.8)	0.82 (0.69, 0.98)	2,364	696 (29.4)	0.80 (0.67, 0.96)
Regular exercise										
	No	2,552	857 (33.6)	1.00 (reference)	2,368	766 (32.4)	1.00 (reference)	2,093	652 (31.2)	1.00 (reference)
	Yes	1,422	528 (37.1)	1.11 (0.95, 1.29)	1,323	472 (35.7)	1.09 (0.93, 1.28)	1,151	375 (32.6)	0.98 (0.83, 1.17)
Antihypertensive use										
	No	3,369	1,085 (32.2)	1.00 (reference)	3,190	990 (31.0)	1.00 (reference)	2,842	835 (29.4)	1.00 (reference)
	Yes	605	300 (49.6)	1.02 (0.83, 1.25)	501	248 (49.5)	1.06 (0.85, 1.33)	402	192 (47.8)	1.02 (0.80, 1.31)
Lipid-lowering drug use										
	No	3,517	1174 (33.4)	1.00 (reference)	3,330	1,067 (32.0)	1.00 (reference)	2,950	889 (30.1)	1.00 (reference)
	Yes	457	211 (46.2)	0.93 (0.74, 1.17)	361	171 (47.4)	1.00 (0.77, 1.29)	294	138 (46.9)	1.06 (0.80, 1.41)
Antidiabetic use										
	No	3,790	1289 (34.0)	1.00 (reference)	-	-	-	-	-	-
	Yes	184	96 (52.2)	0.91 (0.62, 1.34)	-	-	-	-	-	-
FH of hypertension										
	No	2,489	899 (36.1)	1.00 (reference)	2,297	799 (34.8)	1.00 (reference)	2,005	658 (32.8)	1.00 (reference)
	Yes	1,485	486 (32.7)	0.95 (0.81, 1.12)	1,394	439 (31.5)	0.92 (0.78, 1.08)	1,239	369 (29.8)	0.92 (0.77, 1.10)

All variables whose values appear in each column were included in the statistical model for that column. IMT, intima-media thickness; OR, odds ratio; CI, confidence interval; FH, family history; BMI, body mass index; SBP, systolic blood pressure.

**Table 3.** Sensitivity analysis of the association between a family history of diabetes, including those with an unknown family history, and increased carotid IMT

Family history of diabetes^1^	No. of people	People with an increased IMT, n (%)	OR (95% CI)^2^	p-value
All participants				
No	3,094	1,079 (34.9)	1.00 (reference)	
Yes	916	323 (35.3)	1.25 (1.05, 1.50)	0.013
Participants without diabetes				
No	2,906	980 (33.7)	1.00 (reference)	
Yes	811	268 (33.1)	1.24 (1.03, 1.50)	0.027
Participants without diabetes or impaired fasting glucose				
No	2,572	823 (32.0)	1.00 (reference)	
Yes	700	214 (32.6)	1.23 (1.00, 1.51)	0.054
Male				
No	1,079	370 (34.3)	1.00 (reference)	
Yes	330	120 (36.4)	1.29 (0.95, 1.76)	0.099
Female				
No	2,015	717 (35.6)	1.00 (reference)	
Yes	586	212 (36.2)	1.35 (1.08, 1.69)	0.008

IMT, intima-media thickness; OR, odds ratio; CI, confidence interval.

1 Parents who were uncertain whether they had diabetes were considered not to have diabetes.

2 Adjusted for age; sex (except for the males and females groups); body mass index; systolic blood pressure; total cholesterol, triglyceride, and hemoglobin A1c levels; smoking and drinking status; regular exercise; use of antihypertensive, lipid-lowering, and antidiabetic drugs (for all participants, males, and females); and family history of hypertension.

## SUPPLEMENTARY MATERIALS

Supplementary Material 1. General characteristics of study participants by sex

## References

[1] Chatterjee S, Khunti K, Davies MJ (2017). Type 2 diabetes. Lancet.

[2] Schwartz CJ, Valente AJ, Sprague EA, Kelley JL, Cayatte AJ, Rozek MM (1992). Pathogenesis of the atherosclerotic lesion. Implications for diabetes mellitus. Diabetes Care.

[3] Moreno PR, Fuster V (2004). New aspects in the pathogenesis of diabetic atherothrombosis. J Am Coll Cardiol.

[4] Chen R, Ovbiagele B, Feng W (2016). Diabetes and stroke: epidemiology, pathophysiology, pharmaceuticals and outcomes. Am J Med Sci.

[5] Eschwege E, Richard JL, Thibult N, Ducimetière P, Warnet JM, Claude JR (1985). Coronary heart disease mortality in relation with diabetes, blood glucose and plasma insulin levels. The Paris Prospective Study, ten years later. Horm Metab Res Suppl.

[6] Mooradian AD (2009). Dyslipidemia in type 2 diabetes mellitus. Nat Clin Pract Endocrinol Metab.

[7] Goraya TY, Leibson CL, Palumbo PJ, Weston SA, Killian JM, Pfeifer EA (2002). Coronary atherosclerosis in diabetes mellitus: a population-based autopsy study. J Am Coll Cardiol.

[8] Kawamori R, Yamasaki Y, Matsushima H, Nishizawa H, Nao K, Hougaku H (1992). Prevalence of carotid atherosclerosis in diabetic patients. Ultrasound high-resolution B-mode imaging on carotid arteries. Diabetes Care.

[9] Lee WL, Cheung AM, Cape D, Zinman B (2000). Impact of diabetes on coronary artery disease in women and men: a meta-analysis of prospective studies. Diabetes Care.

[10] Arvind K, Pradeepa R, Deepa R, Mohan V (2002). Diabetes & coronary artery disease. Indian J Med Res.

[11] Valdez R, Yoon PW, Liu T, Khoury MJ (2007). Family history and prevalence of diabetes in the U.S. population: the 6-year results from the National Health and Nutrition Examination Survey (1999-2004). Diabetes Care.

[12] Sarlund H, Pyörälä K, Penttilä I, Laakso M (1992). Early abnormalities in coronary heart disease risk factors in relatives of subjects with non-insulin-dependent diabetes. Arterioscler Thromb.

[13] Anderwald C, Stadler M, Golay A, Krebs M, Petrie J, Luger A (2010). Impact of family history on relations between insulin resistance, LDL cholesterol and carotid IMT in healthy adults. Heart.

[14] Kao WH, Hsueh WC, Rainwater DL, O’Leary DH, Imumorin IG, Stern MP (2005). Family history of type 2 diabetes is associated with increased carotid artery intimal-medial thickness in Mexican Americans. Diabetes Care.

[15] Pannacciulli N, De Pergola G, Ciccone M, Rizzon P, Giorgino F, Giorgino R (2003). Effect of family history of type 2 diabetes on the intima-media thickness of the common carotid artery in normal-weight, overweight, and obese glucose-tolerant young adults. Diabetes Care.

[16] Anderwald C, Pfeiler G, Nowotny P, Anderwald-Stadler M, Krebs M, Bischof MG (2008). Glucose turnover and intima media thickness of internal carotid artery in type 2 diabetes offspring. Eur J Clin Invest.

[17] Park GM, Cho YR, Lee SW, Yun SC, Gil EH, Kim DW (2016). Family history of diabetes and the risk of subclinical atherosclerosis. Diabetes Metab.

[18] Harrison TA, Hindorff LA, Kim H, Wines RC, Bowen DJ, McGrath BB (2003). Family history of diabetes as a potential public health tool. Am J Prev Med.

[19] O’Donnell CJ (2004). Family history, subclinical atherosclerosis, and coronary heart disease risk: barriers and opportunities for the use of family history information in risk prediction and prevention. Circulation.

[20] Shim JS, Song BM, Lee JH, Lee SW, Park JH, Choi DP (2019). Cohort profile: the Cardiovascular and Metabolic Diseases Etiology Research Center cohort in Korea. Yonsei Med J.

[21] Shim JS, Song BM, Lee JH, Lee SW, Park JH, Choi DP (2017). Cardiovascular and Metabolic Diseases Etiology Research Center (CMERC) cohort: study protocol and results of the first 3 years of enrollment. Epidemiol Health.

[22] Lee JH, Choi DP, Shim JS, Kim DJ, Park SH, Kim HC (2016). Inter-rater reliability of carotid intima-media thickness measurements in a multicenter cohort study. J Health Info Stat.

[23] Oh JY, Yang YJ, Kim BS, Kang JH (2007). Validity and reliability of Korean version of International Physical Activity Questionnaire (IPAQ) short form. J Korean Acad Fam Med.

[24] Kwak MS, Cho SM, Shim JS, Kim DJ, Youm Y, Kim HC (2020). Association of social network size and composition with physical activity in Korean middle-aged adults. Epidemiol Health.

[25] Yang YJ (2019). An overview of current physical activity recommendations in primary care. Korean J Fam Med.

[26] WHO Expert Consultation (2004). Appropriate body-mass index for Asian populations and its implications for policy and intervention strategies. Lancet.

[27] Malih N, Sohrabi MR, Abadi A, Arshi S (2021). Determinants of adherence to diabetes screening in Iranian adults with a positive family history of diabetes. J Prev Med Public Health.

[28] Mohan V, Shanthirani CS, Deepa R (2003). Glucose intolerance (diabetes and IGT) in a selected South Indian population with special reference to family history, obesity and lifestyle factors--the Chennai Urban Population Study (CUPS 14). J Assoc Physicians India.

[29] Lusis AJ, Mar R, Pajukanta P (2004). Genetics of atherosclerosis. Annu Rev Genomics Hum Genet.

[30] Goldfine AB, Beckman JA, Betensky RA, Devlin H, Hurley S, Varo N (2006). Family history of diabetes is a major determinant of endothelial function. J Am Coll Cardiol.

[31] Trifiletti A, Lasco A, Scamardi R, Cincotta M, Gaudio A, Barbera N (2002). Hemostasis and fibrinolysis factors in first-degree relatives of patients with type 2 diabetes without hypertension. Pathophysiol Haemost Thromb.

[32] Murabito JM, Nam BH, D’Agostino RB Sr, Lloyd-Jones DM, O’Donnell CJ, Wilson PW (2004). Accuracy of offspring reports of parental cardiovascular disease history: the Framingham Offspring Study. Ann Intern Med.

[33] Tang R, Hennig M, Thomasson B, Scherz R, Ravinetto R, Catalini R (2000). Baseline reproducibility of B-mode ultrasonic measurement of carotid artery intima-media thickness: the European Lacidipine Study on Atherosclerosis (ELSA). J Hypertens.

[34] Nathan DM, Davidson MB, DeFronzo RA, Heine RJ, Henry RR, Pratley R (2007). Impaired fasting glucose and impaired glucose tolerance: implications for care. Diabetes Care.

