# Supplementary material for: Association between a family history of diabetes and carotid artery atherosclerosis in Korean adults
Source: Epidemiol Health. 2021 Aug 3;43:e2021049. doi: 10.4178/epih.e2021049 (PMC8510832; doi:10.4178/epih.e2021049)
Supplement: Supplementary Material 1. — General characteristics of study participants by sex [file epih-43-e2021049-suppl.docx]

| **Supplementary Material 1. General characteristics of study participants by sex** | | | | | | | | | | | | | | | | | | | | | | |
| --- | --- | --- | --- | --- | --- | --- | --- | --- | --- | --- | --- | --- | --- | --- | --- | --- | --- | --- | --- | --- | --- | --- |
| Variables | Men (n=1,404) | | | | | | | |  | *p*-value | |  | Women (n=2,570) | | | | | | | | | *p*-value |
|  | Family history of diabetes | | | | | | | |  |  |  |  | Family history of diabetes | | | | | | | | |  |
|  | No (n=1,074) | | | | Yes (n=330) | | | |  |  |  |  | No (n=1,997) | | | | | Yes (n=573) | | | |  |
| Age, years | 50.7 | ± | | 10.2 | 48.3 | ± | | 9.8 | |  | 0.000 | |  | 52.3 | ± | | 8.8 | 50.4 | ± | | 8.8 | <0.001 |
| Body mass index, kg/m² | 24.7 | ± | | 2.8 | 25.3 | ± | | 3.0 | |  | 0.001 | |  | 23.3 | ± | | 3.0 | 23.5 | ± | | 2.9 | 0.204 |
| SBP, mmHg | 124.9 | ± | | 13.9 | 125.0 | ± | | 13.0 | |  | 0.900 | |  | 115.2 | ± | | 14.6 | 114.4 | ± | | 13.4 | 0.224 |
| DBP, mmHg | 80.6 | ± | | 9.9 | 80.7 | ± | | 9.9 | |  | 0.866 | |  | 73.7 | ± | | 9.2 | 73.8 | ± | | 8.5 | 0.832 |
| Total cholesterol, mg/dL | 195.6 | ± | | 34.5 | 195.1 | ± | | 38.0 | |  | 0.819 | |  | 200.1 | ± | | 34.9 | 200.7 | ± | | 37.1 | 0.748 |
| HDL cholesterol, mg/dL | 51.9 | ± | | 13.0 | 49.2 | ± | | 11.9 | |  | 0.000 | |  | 61.1 | ± | | 14.5 | 60.8 | ± | | 14.6 | 0.720 |
| LDL cholesterol, mg/dL | 119.3 | ± | | 32.9 | 118.3 | ± | | 33.5 | |  | 0.607 | |  | 118.3 | ± | | 31.2 | 119.5 | ± | | 33.8 | 0.446 |
| Triglyceride, mg/dL | 126.5 |  | [91–179] | | 139.0 |  | [97–210] | | |  | 0.003 | |  | 98.0 |  | [72–134] | | 97.0 |  | [73–132] | | 0.870 |
| Fasting glucose, mg/dL | 90.0 |  | [84–99] | | 93.5 |  | [85–103] | | |  | 0.000 | |  | 87.0 |  | [81–93] | | 88.0 |  | [82–94] | | 0.002 |
| Hemoglobin A1c, % | 5.7 | ± | | 0.7 | 5.9 | ± | | 1.0 | |  | <0.001 | |  | 5.6 | ± | | 0.6 | 5.7 | ± | | 0.7 | 0.015 |
| Hypertension | 370 |  | (34.5) | | 118 |  | (35.8) | | |  | 0.711 | |  | 436 |  | (21.8) | | 589 |  | (29.5) | | 0.004 |
| Dyslipidemia | 353 |  | (32.9) | | 136 |  | (41.2) | | |  | 0.007 | |  | 93 |  | (16.2) | | 180 |  | (34.4) | | 0.405 |
| Diabetes | 93 |  | (8.7) | | 54 |  | (16.4) | | |  | <0.001 | |  | 91 |  | (4.6) | | 45 |  | (7.9) | | 0.003 |
| Family history of hypertension | 287 |  | (26.7) | | 180 |  | (54.6) | | |  | <0.001 | |  | 697 |  | (34.9) | | 321 |  | (56.0) | | <0.001 |
| Family history of cerebrovascular disease |  |  |  | |  |  |  | | |  |  | |  |  |  |  | |  |  |  | |  |
| Stroke | 155 |  | (14.4) | | 46 |  | (13.9) | | |  | 0.894 | |  | 301 |  | (15.1) | | 87 |  | (15.2) | | 1.000 |
| Myocardial infarction | 56 |  | (5.2) | | 32 |  | (9.7) | | |  | 0.005 | |  | 118 |  | (5.9) | | 61 |  | (10.7) | | <0.001 |
| Medication uses | 186 |  | (17.3) | | 61 |  | (18.5) | | |  | 0.686 | |  | 299 |  | (15.0) | | 59 |  | (10.3) | | 0.005 |
| Antihypertensives | 99 |  | (9.2) | | 38 |  | (11.5) | | |  | 0.261 | |  | 250 |  | (12.5) | | 70 |  | (12.2) | | 0.903 |
| Lipid-lowering drug | 57 |  | (5.3) | | 42 |  | (12.7) | | |  | <0.001 | |  | 53 |  | (2.7) | | 32 |  | (5.6) | | 0.001 |
| Antidiabetics |  |  |  | |  |  |  | | |  |  | |  |  |  |  | |  |  |  | |  |
| Health behaviors |  |  |  | |  |  |  | | |  |  | |  |  |  |  | |  |  |  | |  |
| Current smoker | 359 |  | (33.4) | | 115 |  | (34.9) | | |  | 0.681 | |  | 61 |  | (3.1) | | 16 |  | (2.8) | | 0.853 |
| Current drinker | 918 |  | (85.5) | | 286 |  | (86.7) | | |  | 0.652 | |  | 1,315 |  | (65.9) | | 400 |  | (69.8) | | 0.085 |
| Regular exercise | 423 |  | (39.4) | | 124 |  | (37.6) | | |  | 0.600 | |  | 665 |  | (33.3) | | 210 |  | (36.7) | | 0.150 |
| Carotid IMT (mean), mm | 0.688 | ± | | 0.128 | 0.684 | ± | | 0.127 | |  | 0.628 | |  | 0.674 | ± | | 0.120 | 0.669 | ± | | 0.116 | 0.420 |
| Values are presented as means ± standard deviations, medians [interquartile range], or numbers (%).  SBP, systolic blood pressure; DBP, diastolic blood pressure; HDL, high-density lipoprotein; LDL, low-density lipoprotein; IMT, intima media thickness. | | | | | | | | | | | | | | | | | | | | | | |
|  |  |  |  |  |  |  |  |  |  |  |  |  |  |  |  |  |  |  |  |  |  |  |
